# Frizzled receptors in melanomagenesis: From molecular interactions to target identification

**DOI:** 10.3389/fonc.2022.1096134

**Published:** 2022-12-23

**Authors:** Sheikh A. Umar, Bo Dong, Minakshi Nihal, Hao Chang

**Affiliations:** ^1^ Department of Dermatology, University of Wisconsin-Madison, Madison, WI, United States; ^2^ William S. Middleton Memorial Veterans Hospital, Madison, WI, United States

**Keywords:** frizzled, Wnt/β-catenin signaling, planar cell polarity, cancer, melanoma

## Abstract

Frizzled (FZD) proteins are receptors for the WNT family ligands. Inherited human diseases and genetic experiments using knockout mice have revealed a central role of FZDs in multiple aspects of embryonic development and tissue homeostasis. Misregulated FZD signaling has also been found in many cancers. Recent studies on three out of the ten mammalian FZDs in melanoma have shown that they promote tumor cell proliferation and invasion, *via* the activation of the canonical WNT/β-catenin or non-canonical PCP signaling pathway. In this concise review, we summarize our current knowledge of individual FZDs in melanoma, discuss the involvement of both the canonical and non-canonical pathways, and describe ongoing efforts to target the FZD receptors for melanoma treatment.

## 1 Introduction

Frizzled (FZD) family proteins belong to the G-protein coupled receptors (GPCRs), which are the largest group of cell surface receptors found in humans (1). There are six classes of GPCRs: Rhodopsin family (Class A), secretin receptor family (Class B), metabotropic glutamate/pheromone family (Class C), fungal mating pheromone receptor family (Class D), cyclic AMP receptor family (Class E), and FZD/Smoothened (SMO) family (Class F) (2). Compared to other GPCRs, FZD family receptors are thought to be atypical as the activity level of G protein signaling mediated by FZD is much lower than that of a typical GPCR (3). Spontaneous and targeted mutations in mammalian Frizzled genes have revealed their essential roles in various developmental and homeostatic processes, including palate and heart morphogenesis, tissue polarity, vascular formation and maintenance, and the development of the central nervous system, kidney, and bone (4). Increasing evidence also suggests that FZDs play an important role in cancer development and progression (5).

Frizzled receptors can activate three signaling pathways: canonical WNT/β-catenin pathway, non-canonical planar cell polarity (PCP) pathway, and WNT/calcium signaling pathway (4, 6). The canonical WNT/β-catenin signaling pathway is characterized by the stabilization of β-catenin upon ligand binding (5). The PCP pathway controls the cell/tissue polarity along the body axis, when mutated, results in various developmental defects in the body (7, 8). The WNT/calcium signaling pathway is defined by the regulation of intracellular Ca^2+^ levels and the activation of many calcium-sensitive enzymes (9). Although the activation of these three signaling pathways seems distinct and independent, convergent models of signaling networks where several pathways act in a coordinated and interdependent manner have been proposed (10). The role of Frizzled receptors in development and other cancers has been well-reviewed elsewhere (4, 5, 11). Here, we focus on summarizing our current knowledge of FZDs in melanoma. We describe the role of individual FZDs in melanoma, discuss the involvement of both the canonical WNT/β-catenin and PCP pathways, and highlight potential targeted therapy on the FZD signaling pathways for melanoma treatment.

## 2 FZDs and WNT ligands

**Figure 1:**
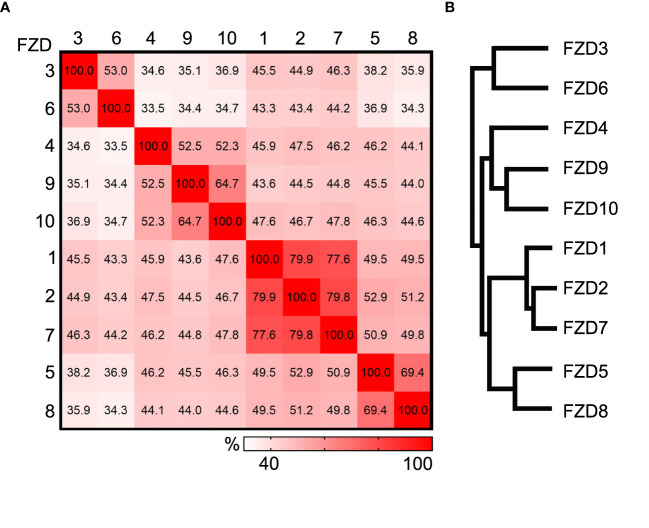
Comparison of human Frizzled proteins. **(A)** Heat plot showing the percent identity matrix of the 10 human Frizzled amino acid sequences. **(B)** Phylogram showing the relatedness of the 10 human FZDs. Sequence compared using the Clustal Omega multiple sequence alignment tool.

There are ten members in the Frizzled family, FZD1-10 in humans and Fzd1-10 in mice. They are between 500 to 700 amino acids in length and can be divided into four sub-families based on the amino acid sequence homology (4). Results of the detailed comparison are shown in **Figure 1** using the Clustal Omega multiple sequence alignment tool (12). In humans, FZD3 and FZD6 share 53% identity, FZD4, FZD9, and FZD10 share 52-65% identity, FZD1, FZD2, and FZD7 share approximately 78-80% identity, and FZD5 and FZD8 share 69% identity. FZDs from different sub-families share 34-53% identity. These numbers are very similar for mouse Frizzled proteins. Common structures of FZDs include an extracellular N-terminus, seven hydrophobic transmembrane domains, and an intracellular C-terminus. The N-terminus contains a conserved 120 amino acids cysteine-rich domain (CRD) connected to the first transmembrane helix by a hydrophilic linker of 70-120 amino acids (13). FZD utilizes CRD as a necessary and sufficient binding site for various ligands, including the WNT proteins, R-spondin, and Frizzled-related proteins (14–17). More precise WNT binding sites on CRD were determined by resolving the crystal structure of CRDs from mouse Fzd8 and secreted Frizzled-related protein 3, and it was shown that CRDs are predominantly α-helical held in place by the disulfide bonds formed by the ten conserved cysteines (18). The co-crystal structure of Xenopus Wnt8 in complex with mouse Fzd8-CRD well explained how a Wnt ligand engages the Fzd receptor (19). The first crystal structure of full-length human FZD has been resolved recently in a ligand-free state at a resolution of ~2 Å, paving the way to for better understanding the function of FZDs (20).

**Figure 2:**
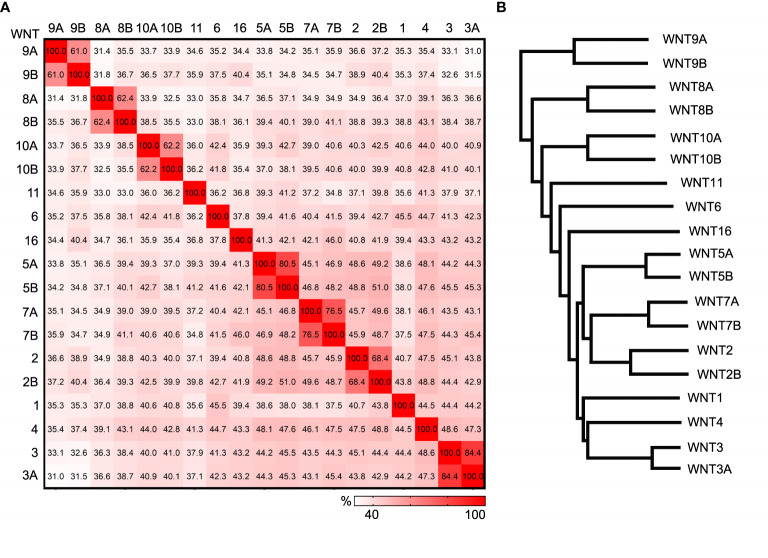
Comparison of human WNT ligands. **(A)** Heat plot showing the percent identity matrix of the 19 human WNT amino acid sequences. **(B)** Phylogram showing the relatedness of the 19 human WNTs. Sequence compared using the Clustal Omega multiple sequence alignment tool.

There are 19 WNT in mammals, and they are typically between 350 to 420 amid acids in length. WNT proteins from the same sub-families share 60-85% identity, and WNTs from different sub-families share about 30-50% identity (**Figure 2**). Like FZDs, WNT proteins are widely expressed in many tissues. For example, the expression of 13 *Wnt* genes and nine *Fzd* genes can be detected in the developing mouse skin (21–23). The 190 possible combinations from 19 WNT ligands and 10 FZDs make the study of the paired role of WNT/FZD extremely difficult. Moreover, the existence of non-WNT ligands for FZD receptors and non-FZD receptors for WNT ligands further complicates the pathway activation. For example, Norrin, a secreted protein encoded by the Norrie disease gene, can bind specifically to the Fzd4 receptor and regulate the angiogenesis and endothelial barrier function (24). Although not related to Wnt, Norrin can mimic Wnt for Frizzled recognition and promote Fzd4 clustering and activation (25). Wnt5a can bind to both Frizzled receptors and receptor tyrosine kinase-like orphan receptors (ROR1 and ROR2). The signaling outputs of Wnt5a often depend on the availability of receptor/coreceptor and the competition of other Wnt ligands binding to receptors (26, 27). It has been reported that Wnt5a can both activate and inhibit canonical Wnt signaling (28). Its binding to the ROR receptors is believed to activate non-canonical PCP signaling (29, 30).

## 3 Canonical WNT/β-Catenin signaling and melanoma

The activation of the canonical WNT signaling pathway involves the inhibition of β-catenin degradation complex, resulting in the accumulation of β-catenin in the cytoplasm and the translocation of β-catenin from the cytoplasm into the nucleus to regulate transcription in combination with LEF/TCF transcription factors (5, 6). The canonical WNT signaling plays an important role in multiple steps of normal melanocyte development, including the migration of its precursor neural crest cell, melanocyte lineage specification, and terminal differentiation (31). β-catenin can directly interact with microphthalmia-associated transcription factor (MITF), a key regulator of melanocytes, to promote melanocyte stem cell proliferation (32). In melanoma, the canonical WNT/β-catenin signaling appears to have a cancer-promoting role, similarly as in many other cancers (33). Mutations in components of the canonical WNT signaling pathway genes, such as *APC*, *AXIN1*, and *CTNNB1*, are found at a frequency of 10%, 2.9%, and 5.9% (34). The pro-cancer role of the canonical WNT/β-catenin signaling in melanoma has been well supported by studies with the *Pten/BRaf* mouse model of melanoma. Silencing the canonical Wnt signaling by β-catenin knockout can slow down tumor cell proliferation and inhibit melanoma metastasis, while activation of the canonical Wnt signaling by β-catenin stabilization can accelerate tumor cell proliferation and melanoma metastasis (35). Activation of the WNT/β-catenin signaling can also promote melanomagenesis by bypassing the oncogen-induced senescence (36–38) or enabling immune evasion (39).

## 4 Non-canonical WNT signaling and melanoma

Most of our knowledge about the role of non-canonical WNT signaling pathway in melanoma came from the studies on WNT5A. Gene expression profiling initially identified that *WNT5A* expression correlated with cell motility and invasiveness of human melanoma cells. This increase in melanoma cell invasion was not related to the canonical WNT signaling activation, as no increase in β-catenin expression or nuclear translocation was observed. Instead, protein kinase C (PKC) activation was dramatically increased upon *WNT5A* overexpression (40). Further studies suggested a phenotypic switch model of melanoma cells, in which the expression of *WNT5A* and *CTNNB1* (encodes β-catenin) dictates the proliferation or invasion status. Cells with high *CTNNB1* and low *WNT5A* are highly proliferative, and cells with low *CTNNB1* and high *WNT5A* are highly invasive (41, 42). Tumor cells need to switch from high-*CTNNB1*, low *WNT5A* to low-*CTNNB1*, high *WNT5A* to initiate metastasis and move away from the primary site. Once they arrive at the site of metastasis, they need to switch back to the proliferative phenotype to colonize at the new location. A recent study shows that WNT5A regulates the expression of the tyrosine kinase receptors AXL and MER to promote tumor dormancy and drive dissemination (43). It remains unclear which receptors WNT5A signals through in regulating the phenotype switch, although evidence for ROR2 and FZD5 both exist (40, 44).

## 5 FZDs and melanoma

### 5.1 FZD1, FZD2 and FZD7

Mouse knockout studies have revealed a critical function of Fzd1/2/7 in palate closure, convergent extension of the neural tube, and heart development (45, 46). Among the ten FZDs, FZD7 is the most studied member in cancer. Overexpression of *FZD7* in cancer cells (e.g., intestinal cancer, hepatocellular carcinoma, and breast cancer) often results in increased cell proliferation and tumor growth (11, 47). Thus, targeted inhibition of FZD7 has been considered as a promising approach for cancer therapy. Limited studies in melanoma also indicate a similar pro-oncogenic role of FZD7. Upregulation of *FZD7* expression in melanoma cells contributes to the drug resistance to the BRAF inhibitor PLX4720 (48). Knockdown of *FZD7* in melanoma cells inhibits the formation of xenograft tumors and metastatic growth in the lung following intravenous injection (49). A recent study has shown that the WNT11-FZD7-DAAM1 axis activates Rho-ROCK1/2-Myosin II in melanoma and plays a crucial role in regulating tumor-initiating potential, local invasion, and distant metastasis formation (50). The role of FZD1 and FZD2 in melanoma has not been investigated.

### 5.2 FZD3 and FZD6

Activation of the canonical WNT signaling can be monitored *in vitro* using HEK293 cells carrying a luciferase reporter under the control of 7 LEF/TCF binding sites (Super TOP-FLASH, STF cells) (24). FZD3 and FZD6 are believed to signal mainly through the non-canonical PCP pathway since they do not activate canonical WNT signaling when cotransfected with many WNTs in the STF cells (45). Knockout studies in mice show that *Fzd3* is required for axon guidance of thalamocortical neurons and spinal sensory neurons (51, 52). *Fzd6* is required in patterning hair follicles and follicle-associated structures (53, 54). Together, Fzd3 and Fzd6 also play redundant roles in neural tube closure and patterning hair cells in the inner ear (55).

Both FZD3 and FZD6 have been found to play a pro-cancer role in melanoma. FZD3 is overexpressed in ~20% of human patient melanoma samples (56). Knockdown of *FZD3* in patient-derived melanoma cells reduces melanoma cell proliferation and progression when engrafted into NSG mice (57). We recently found that FZD6 is also overexpressed in multiple melanoma cell lines and patient tissues. Knockdown or knockout of *FZD6* does not affect cell proliferation but slows down cell invasion. Moreover, knockout of *Fzd6* dramatically reduces lung metastasis in the *Pten/BRaf* mouse model of melanoma (58). It will be interesting to dissect the mechanistic difference between FZD3 and FZD6 in melanoma, as they clearly regulate different aspects of melanoma cell behaviors (FZD3 in cell proliferation and FZD6 in cell invasion).

### 5.3 FZD5 and FZD8

The role of FZD5 and FZD8 in melanoma has not been studied directly. Antibodies against FZD5 have been shown to be able to block melanoma cell migration and invasion *in vitro*, possibly by the inhibition of PKC activity (40). An FZD8-related asialylated antiproliferative factor can inhibit the proliferation of two melanoma cell lines *in vitro*, Hs839T and A375 (59). Due to the caveats of the specificity of the chemical inhibitors or blocking antibodies, further studies are needed to directly determine the function of FZD5 and FZD8 in melanoma.

### 5.4 FZD4, FZD9, and FZD10

FZD4 is believed to signal through the canonical Wnt signaling (4). It binds with WNT7A and WNT7B and non-WNT ligand Norrin (24, 60, 61). They can form a signaling complex with co-receptors LRP5 and TSPAN12 to regulate the vascular formation and maintain the blood-retina barrier and the blood-brain barrier (62, 63). FZD9 plays a role in the development of B-cell, dentate gyrus, and bone (64–66). The role of FZD4 and FZD9 in melanoma has not been studied. Expression of FZD10 has been reported in many cancers, including melanoma. In colon cancer, the cytoplasmic staining of FZD10 increases as the tumor progress. Interestingly, an opposite pattern has been observed in melanoma and gastric cancer, as the cytoplasmic staining of FZD10 is much lower in metastatic tumors than in early-stage tumors (67). The significance of the dynamic expression of FZD10 in melanoma is unknown.

## 6 Therapeutic potential of FZD receptors

GPCRs are important drug targets as they are involved in various diseases. As of 2018, about 34% (n=475) of drugs approved by the Food and Drug Administration (FDA) target 108 GPCR family members (68). Given the general cancer-promoting role of FZDs, several strategies have also been explored to inhibit FZDs for cancer treatment. For example, antibodies targeting the extracellular domain of FZD5, FZD7, and FZD10 have been successfully applied to inhibit the individual FZD-induced tumor growth in pancreatic ductal adenocarcinoma, Wilms’ tumor, and synovial sarcoma, respectively (69–71). The monoclonal antibody OMP-18R5 (vantictumab), initially identified by binding to FZD7, can interact with four other FZD receptors (FZD1, FZD2, FZD5, and FZD8) and block the canonical WNT signaling induced by multiple WNT family members. This antibody can inhibit the growth of a range of tumor types, including lung, breast, colon, and pancreatic tumors in xenograft studies (72). OMP-54F28 (ipafricept), a recombinant fusion protein consisting of the FZD8 CRD and a human IgG1 Fc fragment, has been designed as a decoy receptor to sequester WNTs and prevent them from binding to FZD8 (73). This recombinant protein is effective in inhibiting FZD8-mediated solid tumor growth in preclinical models, although no notable anti-cancer response has been observed in clinical trials (74–76). Similarly, a soluble extracellular peptide of FZD7 has been utilized as a decoy receptor for inhibiting FZD7 in hepatocellular carcinoma cells (77). The idea of disrupting the FZD-induced signaling pathways to treat cancer has also been tried in melanoma (78). FJ9 is a compound designed to disrupt the interaction between the FZD7 and the PDZ domain of Dishevelled. Melanoma cell line LOX treated with FJ9 *in vitro* can inhibit tumor cell growth and cause significant apoptosis. The effects appear to be related to the inhibition of the canonical WNT signaling. Although the *in vivo* effect of FJ9 on melanoma growth has not been tested, it does inhibit the growth of non-small cell lung cancer xenografts in nude mice.

MicroRNA (miRNA)-based gene therapies have shown promise in preclinical studies for cancer treatment, and several have advanced into clinical testing (79). The discovery of various miRNA(s) of FZD receptors has paved the way for designing suitable miRNA-based therapeutic strategies against different cancers, including melanoma. For example, miR-485-5p has been found to target the 3’-untranslated region (3’-UTR) of *FZD7*. It can inhibit melanoma cell invasion and proliferation *in vitro* by suppressing *FZD7* expression, thus providing a promising therapeutic target for melanoma treatment (80). FZD6 expression can be negatively regulated by several miRNAs, such as miR-199a-5p, miR-125b, and miR-20b (81, 82). Given the pro-invasion role recently identified for FZD6 in melanoma, it will be interesting to see if these miRNAs can be used for preventing melanoma progression and metastasis.

## 7 Conclusion

Genetic studies in animal models have revealed critical functions of FZD receptors in embryonic development and adult tissue homeostasis. Increasing evidence also suggests a role of FZDs in many cancers. FZD activation, either *via* the canonical WNT/β-catenin or the non-canonical pathway, appears to have a universal cancer-promoting role in melanoma. Although known at a descriptive level, FZD3, FZD7, and the most recent addition of FZD6 are involved in melanoma cell survival and invasion. The function of the other seven FZDs in melanoma remains to be studied. Perhaps the greatest challenge is to define the role of individual WNT/FZD pairs in melanoma, not only in cell survival and invasion, but also in drug resistance, immune escape, and tumor microenvironment. A detailed, mechanistic understanding of the FZD signaling pathway in melanoma is needed for developing potential therapy. Effort is already underway to target FZD receptors for cancer treatment, including blocking antibodies, peptide inhibitors, small molecule inhibitors, and miRNA-based therapies. Finding an effective but specific targeted therapy on FZDs for melanoma is still a challenging task, given the complexity of the signaling pathway and broad expression of FZDs in many normal tissues. Specific delivery of the drug(s) to melanoma cells will greatly improve the safety and effectiveness of treatment.

## Author contributions

SU and BD wrote the main body of the paper. MN and HC revised the review. HC conceived and directed the idea of the manuscript. All authors contributed to the article and approved the submitted version.
